# Long-Term Outcomes of Mitral Valve Repair with Limited Resection in Active Endocarditis

**DOI:** 10.3390/jcdd13090413

**Published:** 2026-08-25

**Authors:** Zaki Haidari, Iskandar Turaev, Ender Demircioglu, Stephan Knipp

**Affiliations:** Department of Thoracic and Cardiovascular Surgery, West German Heart and Vascular Centre, University Hospital Essen, 45147 Essen, Germany

**Keywords:** infective endocarditis, mitral valve repair, limited resection, cardiac surgery

## Abstract

**Background**: Current guidelines recommend mitral valve (MV) repair over replacement for active native valve endocarditis. However, traditional techniques involving radical debridement and prosthetic patch reconstruction may limit repair feasibility and long-term durability. This study evaluates the long-term clinical outcomes of an alternative approach utilizing a limited resection and non-patch technique in patients presenting with active native MV endocarditis. **Methods**: Consecutive patients with definite active native MV endocarditis who underwent surgical treatment between January 2016 and December 2021 were retrospectively reviewed. From this cohort, all patients managed with MV repair using a limited resection strategy without patch plasty were selected and compared to patients undergoing MV replacement after radical resection. The prespecified primary endpoints were overall mortality, the incidence of recurrent endocarditis, and the rate of reoperation during five-year follow-up. The Cox proportional hazards model was used to identify predictors of overall mortality and adverse events (composite of death, recurrence, and/or reoperation). **Results**: Out of 128 patients with active native MV endocarditis, 75 patients successfully underwent MV repair via the limited resection and non-patch technique and 53 patients received MV replacement. Five-year mortality rates were 43% in the repair group and 52% in the replacement group, *p* = 0.31. Six patients developed recurrent endocarditis in the repair group, while one patient developed it in the replacement group. Reoperation was necessary in five cases in the repair group, while one patient in the replacement group required reoperation due to paravalvular leakage. Surgical strategy was not associated with mortality or adverse events. Staphylococcal endocarditis was a strong predictor of overall mortality (HR: 2.2 (1.3–3.7)) and adverse events (HR: 2.2 (1.0–4.9)). **Conclusions**: A limited resection approach for active native MV endocarditis appears to be a feasible surgical strategy to enhance valve reparability, particularly in non-staphylococcal cases. Conversely, staphylococcal endocarditis remains a potent independent risk factor associated with increased mortality and adverse events.

## 1. Introduction

Surgical intervention for mitral valve (MV) endocarditis remains a high-risk procedure [1]. Current guidelines recommend valve repair as the preferred surgical strategy whenever feasible [2], given that MV repair is associated with significantly superior survival and lower rates of recurrence and reoperation compared to valve replacement [3,4]. Standard surgical repair techniques typically involve radical resection of infected leaflet tissue (including a margin of healthy tissue) combined with patch plasty to reconstruct leaflet defects [5]. Although overall repair rates are increasing, they remain suboptimal at approximately 25%, largely due to concerns regarding the long-term durability of patch materials [6,7,8,9,10]. Limited resection of infected tissue (without resection of a margin of healthy tissue) is an alternative strategy to increase repair feasibility while avoiding the use of patch materials; however, the potential risk of recurrent infection remains a concern. Here, we evaluate the long-term clinical outcomes of MV repair by limited resection strategy compared to mitral valve replacement after radical resection in patients presenting with active native MV infective endocarditis.

## 2. Methods

### 2.1. Patients

This retrospective, single-center study was conducted at a tertiary referral hospital. Patients with definite active native MV IE, diagnosed according to the modified Duke criteria [11], who underwent cardiac surgery between January 2016 and December 2021 were screened for eligibility. Exclusion criteria comprised any history of prior MV intervention or surgery. All patients received appropriate perioperative antibiotic therapy. Prospectively collected perioperative data were retrieved from the institutional electronic health records. Follow-up was completed via outpatient clinic visits or structured telephone interviews to collect data regarding survival and valve-related complications. The study protocol conformed to the ethical principles of the Declaration of Helsinki and the Good Clinical Practice guidelines, and was approved by the institutional ethics committee. Given the retrospective design, the requirement for written informed consent was waived, and no incentives were offered to the participants.

### 2.2. Limited Resection Technique

Baseline cardiac and valvular functions were routinely evaluated via preoperative transesophageal echocardiography. Standard surgical access was through median sternotomy; minimally invasive surgery was usually avoided to reduce cardiopulmonary bypass and aortic cross-clamp times. Following median sternotomy or right lateral thoracotomy and systemic heparinization, venous and arterial cannulation was established. Myocardial protection and cardioplegic arrest were achieved utilizing topical cooling combined with antegrade cold crystalloid cardioplegia (Custodiol, Dr. Franz Koehler Chemie, Bensheim, Germany). The mitral valve was exposed via either a left atriotomy or a transseptal approach, dictated by the requirement for concomitant tricuspid valve intervention. A meticulous valve analysis was conducted to identify infective and degenerative lesions. In mitral valve repair, after removal of all vegetations, macroscopically infected tissue was resected with minimal margins relative to the adjacent healthy tissue. Abscess cavities, when present, were opened to allow complete debridement of necrotic debris, followed by local disinfection with polyvidone and vancomycin. Leaflet defects, including resections and perforations, were repaired via direct closure using interrupted nylon monofilament (Cardionyl^®^, Peters Surgical and Catgut GmbH, Markneukirchen, Germany) sutures. Additional reconstructive techniques comprised chordal transfer or replacement, with the repair finalized by a flexible annuloplasty band using non-pledgeted double-armed 2-0 braided polyester sutures (J&J MedTech, Ethicon, New Brunswick, NJ, USA). In mitral valve replacement, radical resection of all infected tissue was performed. All prostheses and sutures were soaked in vancomycin solution during the operation. Concomitant procedures were performed as indicated for secondary cardiac pathologies. Upon successful weaning from cardiopulmonary bypass, intraoperative transesophageal echocardiography was utilized to assess the surgical result. Postoperatively, patients were transferred to the intensive care unit for hemodynamic and pulmonary monitoring alongside guideline-directed antibiotic therapy. Prior to discharge, transthoracic echocardiography was performed to evaluate cardiac and valvular function.

### 2.3. Endpoint Definition

The three prespecified primary endpoints were overall mortality, the incidence of recurrent endocarditis, and the reoperation rate over the five-year follow-up period. Recurrent endocarditis was defined as any reinfection or relapse of MV endocarditis during the follow-up phase. Reoperation comprised any subsequent surgical intervention involving the MV. An adverse event was defined as death and/or MV endocarditis recurrence and/or MV reoperation and was analyzed as time-to-first-event.

### 2.4. Statistics

Statistical analysis was performed using IBM SPSS Statistics software, version 32.0 (IBM Corp., Armonk, NY, USA). Continuous variables are expressed as means with standard deviations or medians with interquartile ranges, depending on data distribution. Categorical data are presented as absolute numbers and percentages. For the evaluation of imbalances between the two groups, standardized mean difference (SMD) for pre- and intraoperative variables was calculated. An SMD value of less than 0.2 was considered an acceptable difference. The Cox proportional hazards model was used to identify independent predictors of overall mortality and adverse events. Overall and event-free survival rates between MV repair and replacement were compared using Cox regression survival curves, adjusted for confounders (variables with an SMD ≥ 0.2) and for significant predictors of mortality. All statistical tests were two-sided, and a *p*-value < 0.05 was considered statistically significant.

## 3. Results

### 3.1. Baseline Characteristics

**Figure 1:**
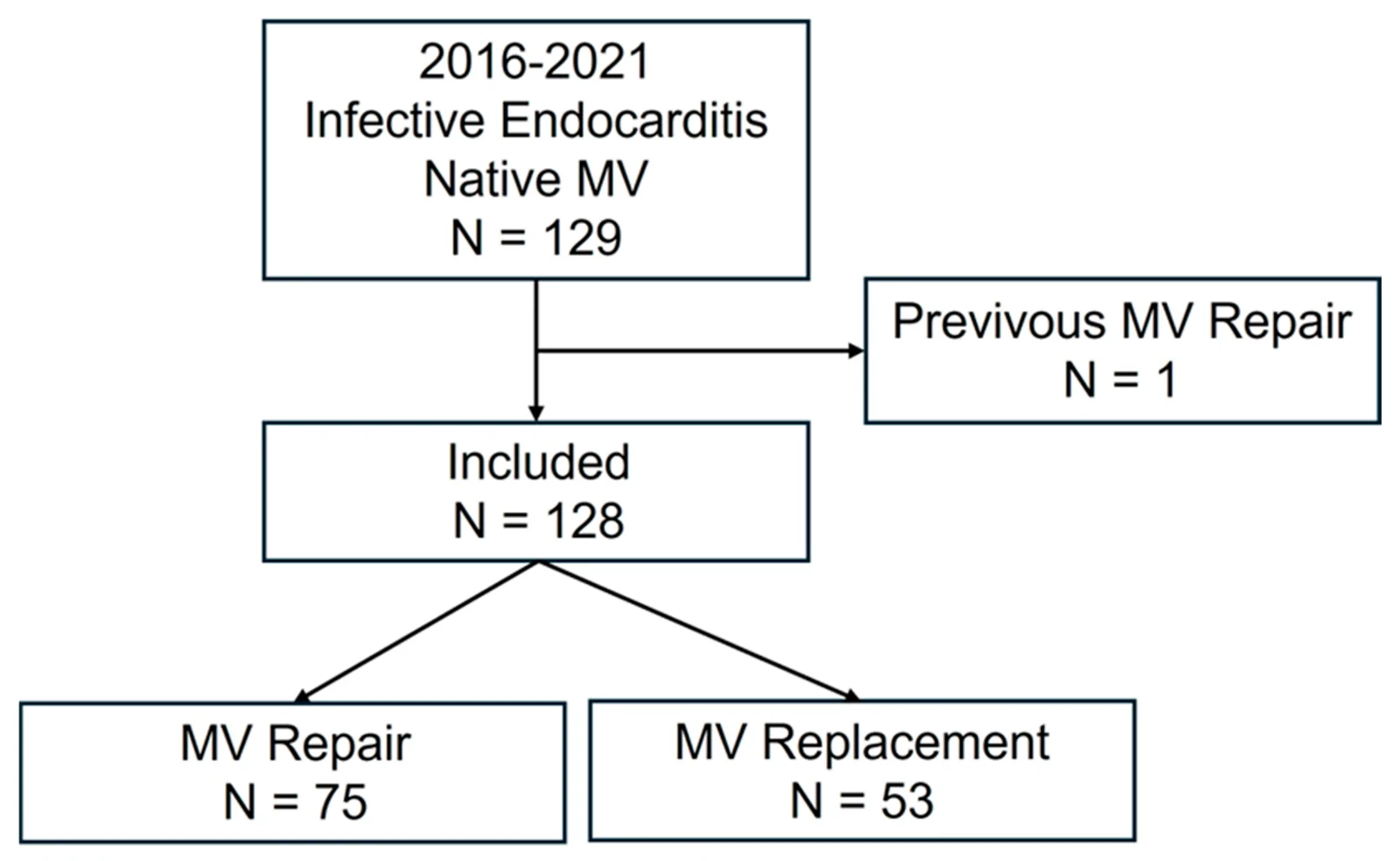
Study flow chart. MV, mitral valve.

In the six-year period (2016–2021), 129 patients with native MV infective endocarditis underwent surgery. Only one patient, who had previous MV surgery for complete atrioventricular septal defect, was excluded from the analysis. A total of 75 patients received MV repair utilizing the limited resection technique, and 53 patients received MV replacement (Figure 1). Preoperative baseline characteristics, clinical status, and echocardiographic parameters are summarized in Table 1. Mean age was 60 years in MV repair and 65 years in MV replacement, *p* = 0.05. The median surgical risk according to EuroSCORE II was 3.22 in the repair versus 5.16 in the replacement groups. The median time from diagnosis to surgery (surgical delay) was comparable between the two groups. Most important baseline characteristics were comparable (SMD < 0.2) between the repair and replacement groups, except for age, EuroSCORE II, and NYHA functional classification III–IV.

### 3.2. Endocarditis Characteristics

The endocarditis characteristics for the entire cohort are summarized in Table 2. The most common causative microorganisms were staphylococcal species. The most frequent indication for surgical intervention was the presence of large or embolized vegetation, followed by heart failure. There were no differences in terms of causative microorganisms and indication for surgery between the repair and replacement groups. Isolated MV surgery was performed on 39 patients (52%) in the repair group, while it was performed on 17 patients (32%) in the replacement group, *p* = 0.03. Concomitant procedures, including coronary artery bypass grafting, aortic valve, and tricuspid valve surgeries, were more often performed in the replacement group (*p* = 0.05). In five cases, a minimally invasive approach was applied. The median cardiopulmonary bypass and aortic cross-clamp times were significantly higher in the replacement group (*p* < 0.01), reaching an SMD of 0.302 and 0.269, respectively.

### 3.3. Endpoints

Five-year follow-up was complete in 96% of the cases, 100% in the MV repair group and 91% in the MV replacement group, *p* = 0.01. The endpoints are presented in Table 3. Thirty-day mortality was 16% in the repair group and 25% in the replacement group. At the five-year follow-up, the overall mortality and adverse event rates rose to 43% and 51% in the repair group versus 52% and 54% in the replacement group, respectively. The hazard ratios for primary endpoints did not differ significantly between repair and replacement groups. Early postoperative complications included mostly revision due to bleeding, respiratory and renal failure requiring reintubation and dialysis. The ICU and hospital stay were comparable between the two groups.

Recurrent endocarditis (Table 4) occurred in seven patients, six in the repair group and one in the replacement group. The causative microorganisms at the index operation in recurrent cases were *Staphylococcus aureus* in three patients, streptococcal species in two cases, Citrobacter koseri in one case, and negative culture in one case. The time intervals between the index operation and recurrence varied between 1 and 19 months; three cases were in the first six months. Five patients (Patient 1–5) with recurrence in the repair group underwent surgical reoperation, which comprised two repeat repairs and three valve replacements. One patient (Patient 6) could be successfully managed with antibiotic therapy. In the replacement group, one patient developed early (after 4 weeks) recurrent endocarditis and was managed medically, and one reoperation was required for paravalvular leakage after 3 months. Notably, all seven patients with recurrent endocarditis were alive at the end of the five-year follow-up period.

### 3.4. Predictors of Mortality and Adverse Events

**Figure 2:**
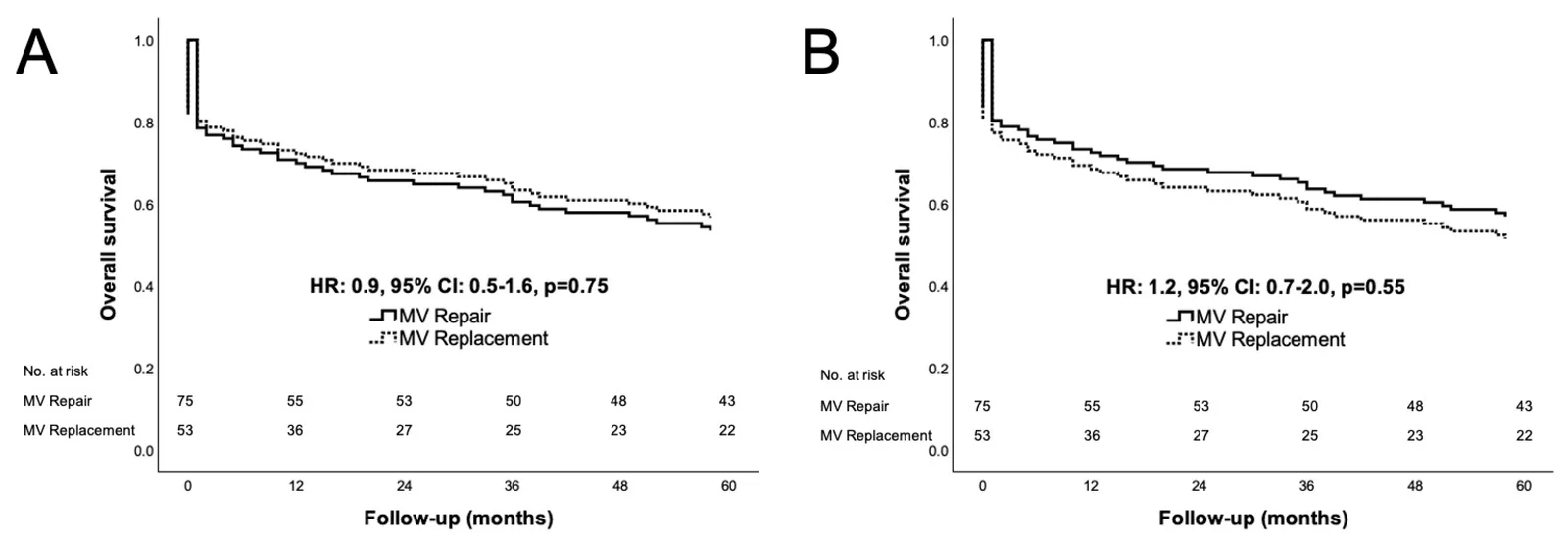
Survival curves showing overall survival of patients with active native mitral valve endocarditis undergoing MV repair and replacement, adjusted for age, EUROScore II, NYHA fc III–IV, and CPB and ACC times (**A**) and for age > 65 years and staphylococcal species as causative microorganism (**B**). MV, mitral valve; HR, hazard ratio; CI, confidence interval. EuroSCORE, European System for Cardiac Operative Risk Evaluation; NYHA fc, New York Heart Association functional classification; CPB, cardiopulmonary bypass; ACC, aortic cross-clamp.

Univariate Cox proportional hazards analysis revealed age >65 years and staphylococcal species as predictors of overall mortality during five-year follow-up. These predictors remained statistically significant in multivariate analysis for overall mortality. Dialysis dependency and staphylococcal species appeared as predictors of adverse events in the univariate Cox proportional hazards model. After multivariate analysis, these predictors remained significant for adverse events. Surgical strategy was not a predictor of overall mortality or adverse events (Table 5). Cox survival curves showed that adjusted overall survival did not differ between MV repair and replacement (Figure 2).

## 4. Discussion

Several studies have evaluated the clinical utility of MV repair in patients presenting with IE. While the majority of evidence demonstrates a distinct survival benefit for MV repair over replacement, significant concerns persist regarding postoperative recurrence and reoperation rates [12,13,14,15,16,17]. Indeed, recent data indicate that relapse rates following MV repair can reach 13%, with virtually all recurrent cases necessitating subsequent surgical intervention [18,19]. Consequently, the complete and meticulous eradication and aggressive debridement of all infected tissue remain paramount to ensure favorable outcomes in cardiac surgery for IE. For cases deemed appropriate for repair, radical resection of the infected valve structures is mandatory to effectively mitigate the risk of recurrent infection. However, a major disadvantage of this radical approach is the creation of extensive tissue defects that require reconstruction using patch materials. To address this challenge, we previously reported the short-term outcomes of a limited-resection strategy compared to the radical-resection approach, finding no significant differences in survival or valve-related complications [20]. Building upon those initial findings, the present study evaluates the long-term clinical outcomes of the limited resection strategy. In the long-term, our current findings support the use of the limited resection strategy to increase repair rates without compromising clinical outcomes.

Within this cohort, the surgical mortality rate for mitral valve (MV) endocarditis reached 16%, aligning closely with European benchmarks [21]. In contrast, a substantial North American registry documented a lower surgical mortality of 10.6% [22]. Although EuroSCORE II predicted a much lower surgical mortality (3.22%), it is well known that EuroSCORE II underestimates surgical mortality in infective endocarditis patients [23]. Regarding long-term outcomes, the existing literature reports mortality rates of up to 35% [24], a figure below the findings in the present study. These variations are primarily driven by patient characteristics such as age, which persist as a critical determinant of survival.

In this study, we found that staphylococcal species as the causative microorganism is a strong predictor of all-cause mortality. This finding has been confirmed by other groups [25,26,27]. While an elevated risk of surgical mortality in staphylococcal endocarditis is well-documented, this study provides the first analysis of a cohort with MV endocarditis. Prior research indicated a three-fold increase in mortality for these patients [28], a trend mirrored by our long-term findings. Notably, the risk of recurrent endocarditis or reoperation remains low between two and five years after MV repair using limited resection. Conversely, prosthetic valve deterioration continues to challenge replacement strategies. Consequently, MV repair should be prioritized whenever feasible.

Regardless of surgical intervention, appropriate antibiotic treatment remains the cornerstone of endocarditis management. Clinically, our institutional protocol aims for a 10-day preoperative antibiotic course whenever feasible. Although early surgery is associated with higher recurrence and/or reoperation rates [29], we could not confirm this in our cohort.

Previous studies have identified preoperative dialysis dependency as an independent predictor of mortality following endocarditis surgery [30,31,32,33]. In the current cohort, 9% of patients required dialysis—a prevalence consistent with prior studies—yet our analysis failed to establish dialysis dependency as a significant predictor of mortality. However, when analyzing the predictors of adverse events, dialysis dependency appeared to be a significant predictor.

## 5. Limitations

Although this single-center retrospective analysis is inherently limited by the absence of a comparable control group and small event numbers, it confirms our previous findings that most valve-related complications occur within the first two postoperative years. As the limited resection strategy was started in 2016, the radical resection technique (before 2016) could not be used as the control group. The lack of data on other risk factors (dental hygiene, drug use) and systematic echocardiographic follow-up further limits the generalizability of the results. However, one-third of cases presented with staphylococcus species, representing a typical cohort of endocarditis patients. Nevertheless, large-scale, prospective randomized controlled trials remain essential to fully evaluate the efficacy of mitral valve repair with limited resection in patients with native mitral valve infective endocarditis.

## 6. Conclusions

MV repair with limited resection and non-patch technique seems to be a feasible surgical option in patients with active native MV infective endocarditis with acceptable long-term outcomes, especially in non-staphylococcal cases. Randomized controlled trials are needed to compare this technique with established strategies.

## Figures and Tables

**Table 1:** Preoperative demographic, clinical, and echocardiographic characteristics.

Variables	MV Repair N = 75	MV Replacement N = 53	SMD	*p*
**Demographics**				
Age, *years ± SD*	60 ± 14	65 ± 13	0.363	0.05
Female, *n (%)*	26 (35)	18 (34)	0.007	0.93
Dialysis-dependent, *n (%)*	7 (9)	5 (9)	0.002	>0.99
Previous cardiac surgery, *n (%)*	3 (4)	7 (13)	0.141	0.16
EuroSCORE II, *% (IQR)*	3.22 (1.97–9.79)	5.16 (2.74–11.82)	0.238	0.15
**Clinical status**				
NYHA fc III–IV, *n (%)*	39 (52)	13 (25)	0.276	<0.01
Preoperative intubated, *n (%)*	7 (9)	5 (9)	0.002	>0.99
Preoperative vasopressor need, *n (%)*	6 (8)	6 (11)	0.085	0.34
Surgical delay, *days (IQR)*	10 (4–21)	9 (5–18)	0.022	0.67
**Echocardiographic parameters**				
LVEF > 50%, *n (%)*	62 (83)	42 (79)	0.043	0.63
Severe MR, *n (%)*	50 (67)	32 (60)	0.065	0.47
Concomitant AV IE, *n (%)*	13 (17)	13 (25)	0.088	0.32
Concomitant TV IE, *n (%)*	6 (8)	5 (9)	0.025	0.76

*Data are presented as mean ± SD or median (interquartile range) and number (percentage); MV, mitral valve; SMD, standardized mean difference; EuroSCORE, European System for Cardiac Operative Risk Evaluation; NYHA fc, New York Heart Association functional classification; LVEF, left ventricular ejection fraction; MR, mitral regurgitation; AV, aortic valve; TV, tricuspid valve; IE, infective endocarditis.*

**Table 2:** Endocarditis characteristics.

Variables	MV Repair N = 75	MV Replacement N = 53	SMD	*p*
**Causative microorganism**				
Staphylococcal species, *n (%)*	27 (36)	21 (40)	0.037	0.68
*Staphylococcus aureus*	21/27	18/21	0.064	0.47
Streptococcal species, *n (%)*	14 (19)	12 (23)	0.049	0.58
Enterococcal species, *n (%)*	8 (11)	7 (13)	0.039	0.66
Others, *n (%)*	5 (7)	3 (6)	0.020	>0.99
Negative culture, *n (%)*	21 (28)	10 (19)	0.105	0.24
**Indication for surgery**				
Large/embolized vegetation, *n (%)*	42 (56)	24 (45)	0.106	0.23
Heart failure, *n (%)*	17 (23)	11 (21)	0.023	0.80
Uncontrolled infection, *n (%)*	10 (13)	15 (28)	0.186	0.04
Valvular disease, *n (%)*	6 (8)	3 (6)	0.045	0.61
**Surgical characteristics**				
Isolated MV surgery, *n (%)*	39 (52)	17 (32)	0.198	0.03
Minimally invasive access, *n (%)*	3 (4)	2 (4)	0.006	>0.99
Concomitant procedure, *n (%)*	36 (48)	34 (64)	0.172	0.05
CABG, *n (%)*	9 (12)	10 (19)	0.095	0.28
AV surgery, *n (%)*	18 (24)	19 (36)	0.129	0.15
TV surgery, *n (%)*	12 (16)	15 (28)	0.149	0.09
CPB time, *minutes (IQR)*	84 (69–114)	118 (85–148)	0.302	<0.01
ACC time, *minutes (IQR)*	53 (44–78)	78 (53–96)	0.269	<0.01

*Data are presented as number (percentage) or median (interquartile range); MV, mitral valve; SMD, standardized mean difference; CABG, coronary artery bypass grafting; AV, aortic valve; TV, tricuspid valve* *; CPB, cardiopulmonary bypass; ACC, aortic cross-clamp.*

**Table 3:** Clinical endpoints.

Variables	MV Repair N = 75	MV Replacement N = 53	HR (95% CI)	*p*
**Primary**				
Mortality (30-Day), *n (%)*	12 (16)	13 (25)	0.80 (0.33–1.94)	0.62
Adverse events (5-Year), *n (%)*	38 (51)	26 (54)	0.94 (0.53–1.66	0.83
Mortality, *n (%)*	32 (43)	25 (52)	0.91 (0.50–1.64)	0.75
Recurrence, *n (%)*	6 (8)	1 (2)	0.16 (0.01–1.7)	0.13
Reoperation, *n (%)*	5 (7)	1 (2)	0.11 (0.01–1.55)	0.10
**Secondary**				
Revision, *n (%)*	6 (8)	4 (8)	NA	>0.99
Stroke, *n (%)*	1 (1)	2 (4)	NA	0.57
Postoperative MCS, *n (%)*	1 (1)	5 (9)	NA	0.08
Reintubation, *n (%)*	8 (11)	11 (21)	NA	0.12
Dialysis, *n (%)*	11 (15)	11 (21)	NA	0.37
ICU stay, *days (IQR)*	4 (2–7)	5 (3–9)	NA	0.49
Hospital stay, *days (IQR)*	12 (8–19)	12 (9–20)	NA	0.89

*Data are presented as numbers (percentages) or medians (interquartile ranges); MCS, mechanical circulatory support; ICU, intensive care unit; MV, mitral valve; HR, adjusted hazard ratio; CI, confidence interval.*

**Table 4:** Analysis of recurrent cases.

Patient	Age, Years	Microorganism	Surgical Strategy	Recurrence Delay, Months
1	42	Strep agalactiae	Repair	2
2	69	Strep salivarius	Repair	19
3	42	Staph aureus	Repair	1
4	52	Staph aureus	Repair	8
5	37	Staph aureus	Repair	15
6	24	Negative culture	Repair	3
7	72	Citerobacter koseri	Replacement	1

**Table 5:** Predictors of overall mortality and adverse events during 5-year follow-up.

	Overall Mortality		Adverse Events	
Variables	Univariate	Multivariate	Univariate	Multivariate
Age > 65 years	2.5 (1.5–4.2)	2.5 (1.5–4.2)	1.6 (1.0–2.6)	
Dialysis dependency	2.0 (0.8–4.1)		2.4 (1.0–5.3)	1.6 (1.0–2.6)
Previous cardiac surgery	2.2 (0.9–5.1)		1.5 (0.6–3.5)	
Surgical delay < 10 days	1.1 (0.6–1.8)		1.0 (0.6–1.7)	
Staphylococcal species	2.2 (1.3–3.7)	2.2 (1.3–3.7)	1.6 (1.0–2.7)	2.2 (1.0–4.9)
Concomitant AV/TV IE	1.5 (0.9–2.6)		1.2 (0.7–1.9)	
MV replacement	1.4 (0.8–2.3)		1.0 (0.6–1.7)	

*Data are presented as hazard ratios (95% confidence intervals); EPV, event per variable; AV, aortic valve; TV, tricuspid valve; IE, infective endocarditis; MV, mitral valve.*

## Data Availability

The data underlying this article will be shared upon reasonable request to the corresponding author.
